# Environmental Enrichment Increases Glucocorticoid Receptors and Decreases GluA2 and Protein Kinase M Zeta (PKMζ) Trafficking During Chronic Stress: A Protective Mechanism?

**DOI:** 10.3389/fnbeh.2015.00303

**Published:** 2015-11-12

**Authors:** Roseanna M. Zanca, Stephen H. Braren, Brigid Maloney, Lisa M. Schrott, Victoria N. Luine, Peter A. Serrano

**Affiliations:** ^1^Department of Psychology, Hunter CollegeCity University of New York, New York, NY, USA; ^2^Department of Pharmacology, Toxicology and Neuroscience, Louisiana State University Health Sciences CenterShreveport, LA, USA; ^3^The Graduate Center of CUNYNew York, NY, USA

**Keywords:** environmental enrichment, stress, corticosterone, GR, GluA2, PKMζ, hippocampus, forced swim test

## Abstract

Environmental enrichment (EE) housing paradigms have long been shown beneficial for brain function involving neural growth and activity, learning and memory capacity, and for developing stress resiliency. The expression of the α-amino-3-hydroxy-5-methyl-4-isoxazolepropionic acid (AMPA) receptor subunit GluA2, which is important for synaptic plasticity and memory, is increased with corticosterone (CORT), undermining synaptic plasticity and memory. Thus, we determined the effect of EE and stress on modulating GluA2 expression in Sprague-Dawley male rats. Several markers were evaluated which include: plasma CORT, the glucocorticoid receptor (GR), GluA2, and the atypical protein kinase M zeta (PKMζ). For 1 week standard-(ST) or EE-housed animals were treated with one of the following four conditions: (1) no stress; (2) acute stress (forced swim test, FST; on day 7); (3) chronic restraint stress (6 h/day for 7 days); and (4) chronic + acute stress (restraint stress 6 h/day for 7 days + FST on day 7). Hippocampi were collected on day 7. Our results show that EE animals had reduced time immobile on the FST across all conditions. After chronic + acute stress EE animals showed increased GR levels with no change in synaptic GluA2/PKMζ. ST-housed animals showed the reverse pattern with decreased GR levels and a significant increase in synaptic GluA2/PKMζ. These results suggest that EE produces an adaptive response to chronic stress allowing for increased GR levels, which lowers neuronal excitability reducing GluA2/PKMζ trafficking. We discuss this EE adaptive response to stress as a potential underlying mechanism that is protective for retaining synaptic plasticity and memory function.

## Introduction

Environmental enrichment (EE) has long been known beneficial for brain function. Such studies support a role for both physical and cognitive activity in reducing neurodegenerative disorders including Alzheimer’s disease and dementia (Laurin et al., 2001; Vaillant and Mukamal, 2001; Valenzuela et al., 2008; Nithianantharajah and Hannan, 2009; Petrosini et al., 2009). Much of this work was developed from early rodent studies identifying that EE-exposed rats developed thicker cortices compared to controls (Diamond et al., 1964; Bennett et al., 1969; Rosenzweig and Bennett, 1969). Later studies contributed to this initial finding with analyses showing morphological changes in dendritic branch length and increased spine densities (Greenough et al., 1973) that are associated with improved learning (Frick and Fernandez, 2003; Leggio et al., 2005; Sampedro-Piquero et al., 2013). In addition to improved learning, EE increases exploratory behavior (Levitsky and Barnes, 1972), including open arm exploration on the elevated plus maze (Fernandez-Teruel et al., 1997; Fernández-Teruel et al., 2002) reflecting reduced anxiety.

EE also has neuroprotective effects against stress involving enhanced hippocampal glucocorticoid receptor (GR) levels (Wislowska-Stanek et al., 2013). Increased GR levels are also associated with improved cognition, and a reduction in motivational and anxiety-related behaviors (Olsson et al., 1994; Reichardt et al., 2000; Fernández-Teruel et al., 2002; Zhang et al., 2013b). Additionally, administration of a GR antagonist or a GR antisense oligonucleotide directly into the hippocampus before learning impaired retention for the forced swim test (FST; De Kloet et al., 1988) and GR-knockout mice show impaired memory consolidation (Oitzl et al., 1997). These results suggest that increases in GR underlie a reduction in fearfulness and anxiety, and contribute to improved learning and memory capacity. Alterations in GR could also allow for better regulation of the hypothalamic-pituitary-adrenal (HPA) axis in an anxious situation and constitute a stress resilience mechanism (Larsson et al., 2002; Fox et al., 2006; Konkle et al., 2010; Hutchinson et al., 2012). However, other studies indicate the opposite effect, showing that GR activation disrupts learning and memory processes (reviewed in Roozendaal, 2002; Joels et al., 2006; Sandi, 2011; Schwabe et al., 2012). These reports suggest that the protective effects against stress-induced cognitive deficits are based on the levels of GR present at the time of stress (Prager and Johnson, 2009). Therefore, we used EE to modulate GR expression and examined the consequences on synaptic trafficking of proteins that are important for long-term memory and synaptic plasticity.

We focus on two markers of synaptic plasticity and memory: the atypical protein kinase M zeta (PKMζ) and the α-amino-3-hydroxy-5-methyl-4-isoxazolepropionic acid (AMPA) receptor subunit GluA2. PKMζ plays a role in long-term memory maintenance across various memory paradigms (Sacktor, 2008, 2011, 2012). The role of PKMζ as necessary for long-term memory function has recently been questioned (Kwapis and Helmstetter, 2014). Results from PKMζ conditional KO mice demonstrate a long-term memory capacity without PKMζ expression (Volk et al., 2013). However, recent data from these same conditional KO mice demonstrate that another atypical kinase, the atypical protein kinase C iota/lambda (PKCι/λ) is upregulated as a compensatory mechanism. It is suggested that PKCι/λ expression allows for long-term memory to persist in the absence of PKMζ (Jalil et al., 2015). PKMζ has also been shown to function in concert with GluA2 during episodes of synaptic plasticity (Ling et al., 2002; Yao et al., 2008) and memory (Migues et al., 2010; Sebastian et al., 2013b). As the trafficking of the GluA2 receptor subunit to the postsynaptic density increases during episodes of synaptic plasticity, clusters of PKMζ/GluA2/PSD95 proteins have been identified (Shao et al., 2012), which prevent the AMPA receptors from undergoing endocytosis (Sacktor, 2011). Stabilizing AMPA receptors within the synaptic membrane is important for memory consolidation (Migues et al., 2010), as well as enhancing the area of mushroom spine heads (mature spines), which are known to increase during fear memory (Sebastian et al., 2013a).

We focus our experiments on identifying the behavioral and biochemical effect of differential housing conditions on both chronic and acute stressors. Our behavioral results show that EE- housed animals show an improved stress response to the FST compared to standard-(ST) housed controls. Our molecular analyses focus on the hippocampal expression of plasma corticosterone (CORT), GR, GluA2, and PKMζ levels after 1 week in the two housing conditions. We focus on these markers since CORT is known to modulate the expression of GR and to increase the trafficking of GluA2 (Groc et al., 2008; Martin et al., 2009; Sarabdjitsingh et al., 2014) and PKMζ (Sebastian et al., 2013a). Our results identify that EE provides an adaptive response to chronic + acute stress that involves the upregulation of GR, but not GluA2 or PKMζ. In contrast, the ST-housed animals show no change in GR but instead increase both GluA2 and synaptic PKMζ after chronic + acute stress. These data identify a potential mechanism for EE-induced stress resilience that could play a significant role in protection against stress-induced cognitive deficits.

## Materials and Methods

### Subjects

Male Sprague-Dawley rats (*n* = 51) from Charles River (Catskill, NY, USA) were purchased at 12 weeks of age (275–325 g). Rats were housed individually at the Hunter College animal facility for 1 week prior to beginning any behavioral assessments. Animal quarters were maintained at constant temperature (22 ± 1°C) and relative humidity (40–50%) with a 12 h light/dark cycle (lights on at 8 am). Food (Harlan Teklad; Frederick, MD, USA) and water were available *ad libitum*.

This study was carried out in accordance with the recommendations of the NIH Guide for the Care and Use of Laboratory Animals developed by the Public Health Service Policy on Humane Care and Use of Laboratory Animals Committee. The protocol was approved by the Hunter College guidelines outlined by the Institutional Animal Care and Use Committee (IACUC).

### Experimental Design

**Figure 1:**
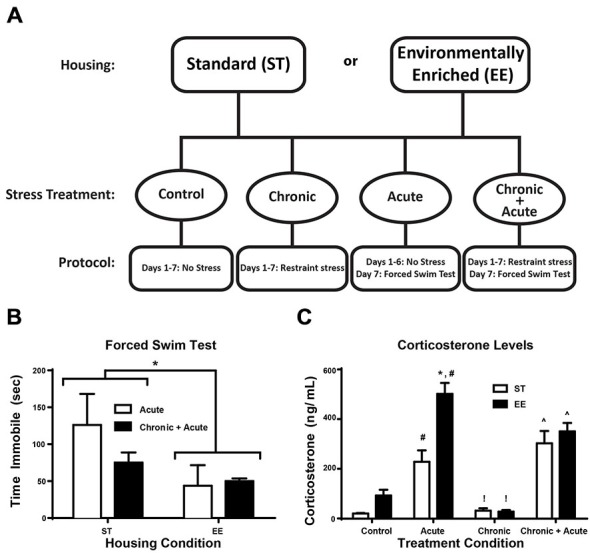
**Environmental enrichment (EE) reduces forced swim immobility time and increases corticosterone (CORT) levels after acute stress. (A)** Experimental design is illustrated across days for the two housing conditions and for the four stress treatments. **(B)** Time immobile during the FST for acute and chronic + acute groups show an overall significant effect of housing condition where EE-housed animals spent significantly less time immobile compared to standard (ST)-housed animals (*p < 0.05). **(C)** There was a significant increase in CORT levels after acute stress for both housing conditions compared to housing matched controls (^#^*p* < 0.05). EE acute animals showed significantly higher CORT compared to ST in the same treatment (*p < 0.01). EE- and ST-housed animals showed a significant reduction in CORT after chronic stress compared to acute stress of the same housing condition (!*p* < 0.01). Chronic + acute stress treatment showed a significant increase in CORT for both ST- and EE-housed animals compared to chronic stress animals of the same housing condition (^∧^*p* < 0.01).

As shown in Figure 1A, the experiment employed a between-groups design comprised of eight independent groups (detailed below). Groups were first divided into two housing conditions: ST or EE. Within each housing condition, there were four stress treatment groups: control (no stress; ST: *n* = 10; EE: *n* = 5), acute (FST only; ST: *n* = 5; EE: *n* = 5), chronic (restraint only; ST: *n* = 11; EE: *n* = 5), and chronic + acute (restraint + FST; ST: *n* = 6; EE: *n* = 4). Each animal was assigned to one of these eight groups.

### Housing Conditions

After 1 week in single housing, rats were assigned to either the ST- or EE-housing condition for 1 week. Rats assigned to the ST condition remained individually housed in standard shoebox cages (48L × 27H × 16W cm) for the duration of the experiment. The EE housing consisted of 10 rats group-housed in a large cage (100L × 70H × 50W cm) that contains two levels connected with ladders and ramps. The EE animals had the addition of chew toys and objects to explore placed in their cage.

### Acute and Chronic Stress

Two stress paradigms were used. The acute stress consisted of a single 10 min FST in a glass cylinder (diameter 20 cm × height 53.3 cm) that was filled with water (21–23°C) to 43 cm, 10 cm below the lip. All FSTs were video-taped and analyzed by researchers blind to the experimental conditions for time immobile using EthoVision XT (Noldus, Netherlands). Chronic stress required each rat to be individually inserted into a restraint tube (Harvard Apparatus, Holliston, MA, USA). Restraint stress occurred for seven consecutive days, 6 h/day. The chronic + acute condition involved seven consecutive days of restraint stress plus an additional acute stress involving the FST within 15 min of being removed from chronic restraint on the 7th day. Trunk blood and hippocampi were collected on the 7th day for all treatment conditions.

### CORT Assay

All subjects were rapidly decapitated immediately after removal from the stressor on the final day. Trunk blood was obtained and brains were removed for hippocampal dissections between 1–3 pm. All blood samples were collected 10 min following the FST or completion of the chronic restraint stress. Blood samples for the control (no stress) condition were collected in the same time window. Blood samples were spun at low speed (3000 g for 10 min at 4°C) to obtain sera for CORT analysis. Following ether extraction of the sera, CORT was analyzed by Enzyme-linked immunosorbent assay (ELISA) kit (Neogen; Lexington, KY, USA). Plates were read in a BioPlex Bead Array Reader (BioRad; Hercules, CA, USA).

### Tissue Fractions

Hippocampi were microdissected and prepared into cytosolic and synaptic fractions as previously reported (Braren et al., 2014). Briefly, tissues were thawed from frozen and homogenized in a TEE (Tris 50 mM; EDTA 1 mM; EGTA 1 mM) buffer containing a SigmaFast, protease inhibitor cocktail (Sigma Aldrich) diluted to contain AEBSF (2 mM), Phosphoramidon (1 μM), Bestatin (130 μM), E-64 (14 μM), Leupeptin (1 μM), Aprotinin (0.2 μM), and Pepstatin A (10 μM). Tissues were homogenized in 200 μl of the TEE-homogenization buffer using 20 pumps with a motorized pestle. Homogenates were transferred to Eppendorf tubes and centrifuged at 3000 g (5 min at 4°C), to remove unhomogenized tissue. The resulting supernatant was centrifuged at 100,000 g for 30 min. After ultracentrifugation, the supernatant was collected and stored as the cytosolic fraction. The remaining pellet was resuspended in 100 μl of homogenizing TEE buffer containing 0.001% Triton X-100, incubated on ice for 1 h and then centrifuged at 100,000 g for 1 h at 4°C. The resulting pellet was resuspended in 50 μl of TEE buffer and stored as the synaptic fraction (Nogues et al., 1994). The Pierce bicinchoninic acid assay (BCA; Thermo Scientific, Rockford, IL, USA) was used to determine protein concentration for each sample. Samples were reduced with 4× Laemmli sample buffer equivalent to 25% of the total volume of the sample and then boiled and stored frozen at −80°C.

### Immunoblots

Samples (20 μg) were loaded onto a Tris/Gly 4–20% midi gel to resolve GAPDH (37 kDa), PKMζ (55 kDa), GluA2 (102 kDa) and GR (98 kDa). Every gel contained 3–4 lanes loaded with the same control sample, all brain sample (ABS). ABS was used to standardize protein signals between gels. Gels were transferred to nitrocellulose membranes in the IBlot^®^ Dry Blotting System (Life Technologies; Carlsbad, CA, USA) for 9 min. Nitrocellulose membranes were then incubated in blocking solution containing 5% sucrose in Tris Buffered Saline with Tween-20 (TBST; 0.1% Tween-20 in TBS) for 30 min at room temperature. Samples were incubated with the following primary antibodies overnight: GluA2 (1:2000; Chemicon, Temecula, CA, USA), GR (1:1000, AbCam, Cambridge, MA, USA), PKMζ (1:2000; Santa Cruz Biotechnology, Santa Cruz, CA, USA); and GAPDH: (1:2000, Chemicon, Temecula, CA, USA). Membranes were washed in TBST for 20 min and probed with Horseradish Peroxidase (HRP) conjugated secondary antibody. Membranes were incubated with Enhanced Chemiluminescence (ECL) substrate and exposed on CL-X Posure Film (Thermo Scientific; Rockford, IL, USA). Films were scanned for quantification with NIH Image J (Rasband, 2014).

### Statistics

For behavioral and molecular analyses, a two-way ANOVA (housing conditions by stress treatments) was used (Prism GraphPad 6.0 Statistical Package, La Jolla, CA, USA). All *post hoc* analyses used Bonferroni-corrected *t*-tests.

## Results

Figure 1A illustrates the experimental design for the two housing conditions and four stress treatments. Figure 1B shows the two-way ANOVA between housing conditions for time immobile during FST for the acute and chronic + acute groups, identifying an overall significant effect of housing [*F_(1,11)_* = 5.17, *p* < 0.05, *n* = 3–6/group]. EE-housed animals spent significantly less time immobile compared to ST-housed animals. There were no significant *post hoc* tests.

In Figure 1C a two-way ANOVA for CORT identifies an overall significant effect of housing [*F*_(1,30)_ = 14.47, *p* < 0.01], treatment [*F*_(3,30)_ = 46.97, *p* = 0.0001], and an interaction [*F*_(3,30)_ = 5.608, *p* < 0.01; *n* = 3–6/group]. *Post hoc* tests show acute stress significantly increased CORT for both ST- and EE-housed animals compared to controls of the same housing condition [ST: *t*_(30)_ = 3.72, *p* < 0.05; EE: *t*_(30)_ = 8.451, *p* < 0.01]. EE animals after acute stress mounted a significantly higher CORT response compared to acute stress ST animals [*t*_(30)_ = 5.65, *p* < 0.01]. Although there was a significant difference in the levels of CORT mounted between EE- and ST-housed animals after acute stress, both conditions showed a significant reduction in CORT after chronic stress compared to their respective acute stress treatments [ST: *t*_(30)_ = 4.24, *p* < 0.01; EE: *t*_(30)_ = 9.793, *p* < 0.01]. After chronic stress, the levels of CORT were not significantly different compared to controls for both housing conditions, suggesting that chronic stress produced an adaptive response to stress. However, both ST- and EE-housed animals treated with chronic + acute stress showed a significant increase in CORT compared to their respective chronic stress treatment [ST: *t*_(30)_ = 6.13, *p* < 0.01; EE: *t*_(30)_ = 5.78, *p* < 0.01]. There were no significant differences between EE- and ST-housed animals after chronic + acute stress.

**Figure 2:**
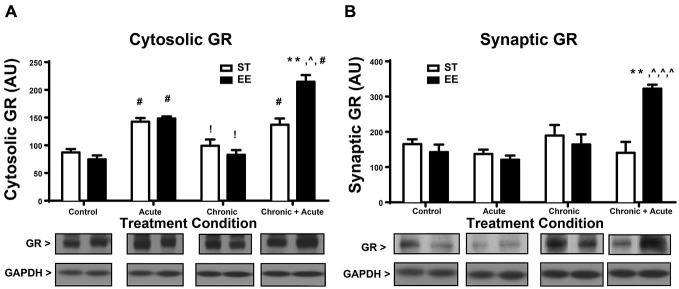
**Environmental enrichment increases glucocorticoid receptor (GR) levels after chronic + acute stress. (A)** Cytosolic GR increased significantly for both ST- and EE-housed animals after acute stress compared to controls of the same housing condition (^#^*p* < 0.01). Both ST- and EE-housed animals showed a significant decrease in cytosolic GR levels after chronic stress compared to acute stress treatment of the same housing condition (!*p* < 0.01). Chronic + acute stress in EE-housed animals showed a significant increase in cytosolic GR compared to ST-housed animals for the same treatment (**p < 0.01) and compared to chronic stress for EE housed animals (^∧^*p* < 0.01). Chronic + acute stress for both ST- and EE-housed animals significantly increased cytosolic GR compared to controls in the same housing conditions (^#^*p* < 0.01). **(B)** Synaptic GR significantly increased after chronic + acute stress for EE housed animals compared to all other treatments within the same housing condition (^∧^*p* < 0.01) and compared to ST chronic + acute (**p < 0.01).

A two-way ANOVA for GR levels in the cytosol (Figure 2A) shows a significant overall effect of housing [*F*_(1,32)_ = 4.73, *p* < 0.05], treatment [*F*_(3,32)_ = 51.87, *p* < 0.01] and an interaction [*F*_(3,32)_ = 12.23, *p* < 0.0; *n* = 4–6/group]. *Post hoc* tests show a significant increase in cytosolic GR for both ST- and EE-housed animals after acute stress compared to their respective controls [ST: *t*_(32)_ = 4.444, *p* < 0.01; EE: *t*_(32)_ = 5.921, *p* < 0.01]. This significant increase in cytosolic GR reflects the internalized receptors, upon binding to CORT, that are released during acute stress treatment. After chronic stress both ST- and EE-housed animals showed a significant decrease compared to their respective acute stress treatment [ST: *t*_(32)_ = 3.63, *p* < 0.05; EE: *t*_(32)_ = 5.27, *p* < 0.01]. This pattern is also observed in CORT levels between acute and chronic treatments (Figure 1C), suggesting that as CORT levels decrease so do the cytosolic levels of GRs, reflecting internalization. Additionally, levels of synaptic GR (Figure 2B) after chronic stress for both housing conditions were not significantly different from their respective controls. This pattern again parallels that of CORT levels between control vs. chronic stress (Figure 1C). Chronic + acute stress in EE animals show a significant increase in cytosolic GR compared to ST chronic + acute animals [*t*_(32)_ = 5.88, *p* < 0.01] and compared to EE chronic animals [*t*_(32)_ = 9.992, *p* < 0.01]. The ST condition did not produce significant *post hoc* differences between chronic + acute and chronic stress conditions. Both ST- and EE-housed animals after chronic + acute stress show a significant increase in cytosolic GR compared to their respective controls [ST: *t*_(32)_ = 4.0, *p* < 0.01; EE: *t*_(32)_ = 10.6, *p* < 0.01]. This result indicates that ST-housed animals show increased cytosolic GR after acute stress and after acute + chronic stress, but not after chronic stress. EE-housed animals show a significantly higher level of cytosolic GR after chronic + acute stress compared to ST chronic + acute, suggesting that chronic stress may serve to upregulate GRs in the EE condition.

A two-way ANOVA for synaptic GR (Figure 2B) shows an overall significant effect of treatment [*F*_(3,25)_ = 6.376, *p* < 0.01] and an interaction [*F*_(3,25)_ = 8.628, *p* < 0.01; *n* = 3–5/condition]. *Post hoc* analyses show that synaptic GR significantly increased after chronic + acute stress for EE-housed animals compared to all other EE treatments [EE control: *t*_(25)_ = 4.8, *p* < 0.01; EE acute: *t*_(25)_ = 5.06, *p* < 0.01; EE chronic: *t*_(25)_ = 4.24, *p* < 0.01]. Synaptic GR in the EE-housed animals for the chronic + acute treatment significantly increased compared to ST chronic + acute [*t*_(25)_ = 5.108, *p* < 0.01]. These data show that EE-housed animals have increased levels of both cytosolic and synaptic GR after chronic + acute stress compared to ST chronic + acute animals.

**Figure 3:**
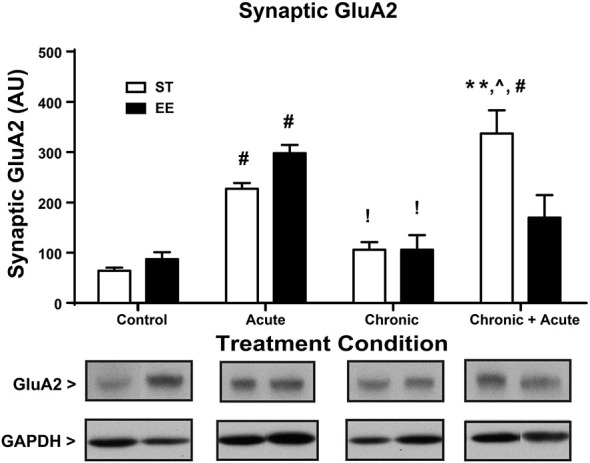
**Environmental enrichment increases GluA2 levels after chronic + acute stress.** ST acute and EE acute significantly increased synaptic GluA2 compared to ST and EE controls in the same housing condition (^#^*p* < 0.01). EE chronic and ST chronic significantly decreased synaptic GluA2 compared to the EE acute and ST acute animals of the same housing condition (!*p* < 0.01). ST chronic + acute significantly increased GluA2 compared to ST chronic (^∧^*p* < 0.01), ST controls (^#^*p* < 0.01) and EE chronic + acute (**p < 0.01).

Figure 3 shows a two-way ANOVA for synaptic GluA2 levels, identifying an overall significant effect of treatment [*F*_(3,29)_ = 27.86, *p* < 0.01] and a significant interaction [*F*_(3,29)_ = 7.872, *p* < 0.01; *n* = 3–7/condition]. *Post hoc* analyses show that acute stress significantly increased the levels of synaptic GluA2 in both ST- and EE-housed animals compared to controls in the same housing condition [ST: *t*_(29)_ = 5.144, *p* < 0.01; EE: *t*_(29)_ = 5.10, *p* < 0.01]. As a consequence of chronic stress, however, both chronic EE and ST animals show a significant decrease in GluA2 levels compared to the acute EE and ST stressed animals in the same housing condition [ST: *t*_(29)_ = 3.543, *p* < 0.05; EE: *t*_(29)_ = 4.858, *p* < 0.01]. GluA2 levels after chronic stress for both ST- and EE-housed animals were not significantly different from their respective controls. *Post hoc* tests show ST chronic + acute stress animals significantly increased GluA2 compared to ST chronic stress [*t*_(29)_ = 6.75, *p* < 0.01], ST controls [*t*_(29)_ = 8.6, *p* < 0.01], and compared to EE chronic + acute [*t*_(29)_ = 4.23, *p* < 0.01]. These data suggest that EE in the chronic condition upregulated GR levels that are trafficked into the synaptic membrane during the chronic + acute stress condition. The upregulation of GR appears to prevent the increase in synaptic GluA2 after chronic + acute stress observed in the ST condition.

**Figure 4:**
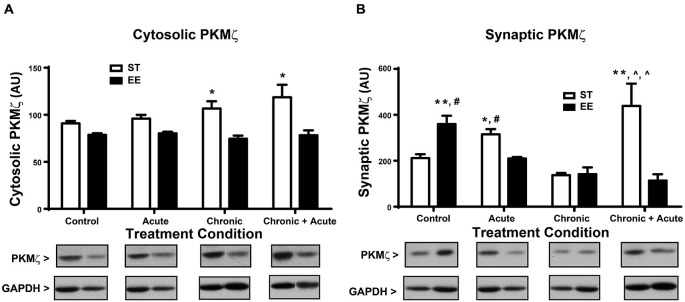
**Environmental enrichment decreases PKMζ levels after acute stress and after chronic + acute stress. (A)** ST chronic is significantly increased compared to EE chronic (*p < 0.05) and ST chronic + acute is significantly increased compared to EE chronic + acute (*p < 0.05). **(B)** ST chronic + acute stress significantly increased synaptic PKMζ levels compared to EE chronic (^∧^*p* < 0.01), ST chronic (^∧^*p* < 0.01) and EE chronic + acute (**p < 0.01). EE control significantly increased synaptic PKMζ compared to ST control (**p < 0.01) and compared to EE acute (^#^*p* < 0.01). ST acute had significantly increased synaptic PKMζ compared to EE acute (*p < 0.05) and compared to ST control (^#^*p* < 0.05).

For the analysis of cytosolic PKMζ levels (Figure 4A), a two-way ANOVA shows an overall significant effect of housing [*F*_(1,43)_ = 22.62, *p* < 0.01; *n* = 4–11/condition]. *Post hoc* analyses show ST chronic significantly increased cytosolic PKMζ compared to EE chronic [*t*_(43)_ = 3.34, *p* < 0.05] and ST chronic + acute significantly increased cytosolic PKMζ compared to EE chronic + acute [*t*_(43)_ = 3.52, *p* < 0.05]. Figure 4B shows the analysis of synaptic PKMζ levels. A two-way ANOVA analysis shows an overall significant effect of housing [*F*_(1,24)_ = 5.745, *p* < 0.05], treatment [*F*_(3,24)_ = 5.527, *p* < 0.01] and an interaction [*F*_(3,24)_ = 11.80, *p* < 0.01; *n* = 3–5 per condition]. *Post hoc* tests show that ST chronic + acute stress significantly increased synaptic PKMζ levels compared to EE chronic [*t*_(24)_ = 5.24, *p* < 0.01], ST chronic [*t*_(24)_ = 5.61, *p* < 0.01] and EE chronic + acute [*t*_(24)_ = 5.32, *p* < 0.01].

The synaptic PKMζ effects observed after chronic stress and chronic + acute stress mirror the effects observed with synaptic GluA2. These data collectively show that ST chronic + acute stress increased synaptic PKMζ and GluA2 levels together with a concomitant decrease in synaptic GR. The opposite pattern emerged in EE chronic + acute stress, which decreased synaptic PKMζ and GluA2 levels, but increased synaptic GR. These data suggest that EE can modulate the expression of GR in the context of stress, which prevents the mobilization of synaptic PKMζ and GluA2. Finally, *post hoc* analyses of synaptic PKMζ levels for the control and acute stress conditions show that EE control has significantly increased synaptic PKMζ compared to ST control [*t*_(21)_ = 4.502, *p* < 0.01] and compared to EE acute [*t*_(21)_ = 4.08, *p* < 0.01]. Additionally, ST acute has significantly increased synaptic PKMζ compared to EE acute [*t*_(21)_ = 3.20, *p* < 0.05] and compared to ST control [*t*_(21)_ = 3.63, *p* < 0.05].

## Discussion

### EE Decreases Depressive-Like Behavior and Increases CORT Acute Stress

One week of EE produced a significant decrease in time immobile during the FST compared to ST-housed animals, indicating that EE animals show more resilience to an acute stressor. These data suggest that EE has protective effects on both acute and chronic stress consistent with the literature (Fox et al., 2006; Llorens-Martin et al., 2007; Brenes et al., 2009; Schloesser et al., 2010; Lehmann and Herkenham, 2011). The EE-housed animals also show significantly higher levels of CORT after the acute stress (FST), which appears inconsistent with some studies. EE has been reported to lower plasma CORT under stress conditions (Mlynarik et al., 2004; Moncek et al., 2004). Several studies have shown that brain levels of CORT do not consistently mirror plasma levels (Lengvari and Liposits, 1977; Croft et al., 2008; Garrido et al., 2012) and may also apply to our data. However, other studies have shown that CORT can enhance learning (Roozendaal et al., 2006), protect against memory deficits (Ebada et al., 2014) and improve memory retention induced by FST (Yuen et al., 2009). Together these studies suggest that EE + acute stress may elevate CORT that reflects protective effects induced by enrichment.

CORT may also be elevated in the EE + acute stress condition as a consequence of the physical demands of the FST (Buwalda et al., 2012). Since the EE animals spend less time immobile, the additional physical activity could contribute to increased CORT. Alternatively, EE housing is mildly anxiogenic as exposure to novelty and physical stimulation may amplify CORT (Adriani and Laviola, 2000; Moncek et al., 2004; Lin et al., 2011).

Thus, it is unknown the degree to which higher CORT levels in EE animals after acute stress may contribute to stress resilience. In contrast to the CORT response after acute stress, chronic restraint stress significantly lowers CORT in both housing conditions, which is consistent with other reports (Sanchez et al., 1998; Djordjevic et al., 2009). Basal CORT levels between EE- and ST-housed animals were not different. In some studies, basal CORT is increased in EE rats (Lin et al., 2011). These discrepancies can be attributed to different lengths of time in the EE conditions or differences in bedding (Heidbreder et al., 2000) and could also reflect differences in the GR levels after stress treatment which would allow for faster negative feedback of CORT (Droste et al., 2009; Wislowska-Stanek et al., 2013).

### EE with Chronic Stress Upregulates GR

**Figure 5:**
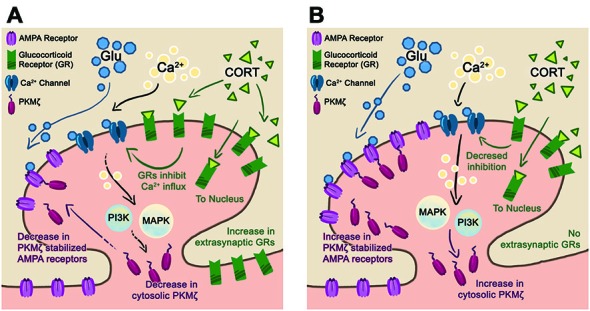
**Proposed synaptic model for stress resilience. (A)** Schematic of a post-synaptic neuron in the EE-housing condition after chronic + acute stress. In this condition, chronic stress upregulates the extra-synaptic GR that are trafficked into the synapse during the subsequent acute stress. The elevated levels of GR reduce Ca2^+^ influx and downstream PI3K, and MAPkinase activates reducing PKMζ/GluA2 trafficking. We hypothesize that maintaining basal levels of synaptic PKMζ/GluA2 during episodes of stress are beneficial towards preserving synaptic plasticity and memory. **(B)** In the ST-housing condition, chronic + acute stress does not upregulate GR levels allowing for increased Ca2^+^ influx and subsequent PKMζ/GluA2 trafficking. We hypothesize that PKMζ/GluA2 trafficking during episodes of stress reduce synaptic plasticity and memory.

Characterizing GR protein expression in the cytosolic and synaptic fractions allows for a clearer understanding of GR trafficking during stress (Lupien and McEwen, 1997; Chen and Qiu, 2001; Djordjevic et al., 2009). Our results show that the cytosolic GR, which reflects internalized receptors, significantly increased after acute stress in both housing conditions. While EE animals did mount a higher CORT response after acute stress than ST-housed animals, no differences in the level of cytosolic GR between housing conditions were found. This finding may be due to sensitivity of the protein assay or rapid trafficking of GR from the cytosol to the nuclear compartment (Djordjevic et al., 2009; Komatsuzaki et al., 2012; Ooishi et al., 2012). After chronic stress, when CORT is at basal levels for both housing conditions, there is a concomitant decrease in cytosolic GR as compared to cytosolic GR levels after acute stress. This change reflects the continuity between CORT and internalized (cytosolic) GR levels. Chronic + acute stress treatment shows that EE-housed animals have increased cytosolic and synaptic GR levels suggesting an upregulation of GR. We believe that there is insufficient time for *de novo* protein synthesis of GR and speculate that during chronic stress treatment GR upregulates in the extra-synaptic membrane (Figure 5), which is not reflected in the cytosolic or synaptic compartments (Ooishi et al., 2012). This interpretation may also explain why we found no differences in GR levels between housing conditions after chronic stress in either the cytosolic or synaptic compartments.

EE increases GR expression in the hippocampus (Olsson et al., 1994; Zhang et al., 2013b) and can increase CORT sensitivity by suppressing the release of corticotropin-releasing factor (CRF) through the negative feedback loop extending from the dorsal hippocampus to the hypothalamus (Antoni, 1986; Fink, 1997). Hence, increasing extra-synaptic GRs in the context of stress may enhance trafficking of GRs to the synapse, resulting in faster decreases in CORT and an increase in cytosolic GR (Reichardt et al., 2000). Additionally, increased GRs are believed to contribute to reducing anxiety characterized by attenuated adrenocortical responses during stress (Lin et al., 2011; Sampedro-Piquero et al., 2014), which is consistent with our FST data and with the effects observed in the transgenic overexpressing G (Reichardt et al., 2000). Previous studies also indicate that EE increases GR expression in specific hippocampal subfields (Vivinetto et al., 2013). Together, these data suggest that EE, which results in increased GR trafficking, reduces behavioral signs of anxiety and depression.

While our results show that GR density increases with EE it remains to be determined whether GR sensitivity to CORT is altered in our paradigm. Animal models of post-traumatic stress disorder (PTSD) show both a decrease GR density with corresponding increase in GR sensitivity after stress (Zoladz et al., 2012). FKBP5 protein and mRNA levels are known to modulate GR sensitivity to CORT (Denny et al., 2000; Scammell et al., 2001; Westberry et al., 2006). Increasing FKBP5 results in GR resistance to CORT that decreases the negative feedback efficiency of the HPA and results in prolonging the stress effect (Binder, 2009). Thus, understanding the degree to which GR density and its sensitivity are altered could have direct implications on PTSD where GR sensitivity is dysregulated (Yehuda et al., 2004; Binder et al., 2008; Ising et al., 2008; Binder, 2009; Heim and Nemeroff, 2009).

### EE Decreases Stress-Induced GluA2 Trafficking

After acute stress, both housing conditions produce an increase in synaptic GluA2 expression, which is consistent with other literature identifying increased GluA2 as a consequence of acute stress (Sebastian et al., 2013a), or CORT treatment in cell culture (Groc et al., 2008; Martin et al., 2009) and hippocampal slices (Sarabdjitsingh et al., 2014). The rapid mobilization of GluA2 observed suggests a CORT/GR-dependent effect. After chronic stress, when both EE- and ST-housed animals show basal levels of CORT, GluA2 levels decrease compared to levels after acute stress, suggesting that synaptic expression is trafficked when GR is active. After chronic + acute stress, ST-housed animals show a significant increase in GluA2 with a concomitant decrease in GR compared to EE. Conversely, EE-housed animals show the opposite pattern, displaying a significant decrease in GluA2 with a concomitant increase in GR. Thus, when synaptic GR levels are elevated, as in the EE condition, trafficking of GluA2 remains at basal levels and is consistent with what is known about GR regulation of voltage-gated calcium and potassium channels, and glutamate-gated AMPA and NMDA receptors. Interacting with these channels and receptors, GR can induce rapid changes to synaptic transmission and neuron excitability (ffrench-Mullen, 1995; Karst et al., 2005; Olijslagers et al., 2008). More specifically, GRs decrease neuronal excitability through L and N calcium channels (ffrench-Mullen, 1995), which decrease the trafficking of GluA2, as it is calcium dependent. The reduction in GluA2 trafficking observed in the EE condition may also result from a decrease in NMDA receptor activation, as CORT indirectly inhibits NMDA-dependent calcium influx (Sato et al., 2004). These data are consistent with the notion that stress response characteristics involving AMPA receptor trafficking may be dependent on GR levels in addition to CORT dose (Prager and Johnson, 2009). Thus, increases in synaptic GR levels result in reduced neuronal excitability and the trafficking of GluA2.

Based on previous literature, we speculate that depressing or maintaining basal levels of GluA2 during episodes of stress is protective for synaptic plasticity and memory function. Recent work shows that the majority of AMPA receptors incorporated into synapses during long-term potentiation (LTP) are from membrane trafficking into the synapse while exocytosed receptors replenish the extra-synaptic pool available for subsequent bouts of plasticity (Newpher and Ehlers, 2008; Makino and Malinow, 2009). This relationship highlights the importance of AMPA receptor trafficking during learning. Conversely, studies also show that CORT can induce the trafficking of AMPA receptors on the plasma membrane (Krugers et al., 2010; Chaouloff and Groc, 2011) and subsequently disrupt LTP (Karst and Joëls, 2005; Alfarez et al., 2006; Sarabdjitsingh et al., 2014). Behaviorally, acute stress-induced increases in GluA2 produce deficits in spatial memory retrieval (Sebastian et al., 2013a). Recent studies show when AMPA receptors are stabilized within the membrane, the negative effects of AMPA receptor mobilization during stress are blocked (Zhang et al., 2013a). Our EE animals show significantly less GluA2 mobilization after chronic + acute stress compared to ST-housed animals, suggesting that the EE paradigm may have a protective effect against stress-induced cognitive deficits. This protective effect may not be entirely driven by stabilizing AMPA receptors in the membrane, but may also involve an increase in GR levels. The mechanism whereby EE induces GR expression is uncertain. It is hypothesized that EE increases monoaminergic activity underlying the increased GR levels and thereby modulates hippocampal function and behavior (Rasmuson et al., 1998; Del Arco et al., 2007). Together, these data show that EE housing produces adaptive responses to chronic stress that may be beneficial in subsequent episodes of hippocampal-dependent plasticity and memory.

### EE Decreases Stress-Induced PKMζ

ST-housed animals show a significant increase in PKMζ after acute stress compared to controls as reported previously (Sebastian et al., 2013a). EE-housed animals show the opposite effect suggesting that separate mechanisms may modulate the effects of acute stress. After chronic + acute stress the synaptic PKMζ level is significantly increased in ST-housed but not EE animals, which is consistent with the increase in GluA2 observed in the ST-housed animals as PKMζ facilitates trafficking of PKMζ in LTP (Yao et al., 2008), in the maintenance of memory (Migues et al., 2010) and during episodes of stress (Sebastian et al., 2013a). These data suggest that synaptic PKMζ increases along with GluA2 when GR levels are not elevated. We speculate that GR levels may regulate GluA2/PKMζ trafficking during stress following an inverse relationship. While the exact mechanism involved for stress-induced increases in PKMζ is unknown, GR activation increases MAPK activity in the hippocampus, which could explain the increase in stress-induced PKMζ levels (Revest et al., 2005). GR also decreases calcium influx (ffrench-Mullen, 1995) explaining the decrease in stress-induced PKMζ levels. Based on these reports, we speculate that significantly increasing levels of GR could switch off or reduce stress-induced trafficking of PKMζ while basal levels of GR allow for stress-induced mobilization of PKMζ (Figure 5). Other reports identify GR-dependent increases in large spines (Komatsuzaki et al., 2012), which is consistent with reports showing both increases in PKMζ and GluA2 within mushroom spines after acute stress (Sebastian et al., 2013a).

## Conclusion and Clinical Implication

Consistent with previously discussed reports, EE has neuroprotective effects against stress. Our results identify better forced swim performance (less time immobile) in EE-housed animals compared to ST-housed animals and altered expression of various synaptic markers. The GR receptor is differentially trafficked and expressed as a consequence of housing. The EE-housed animals demonstrate higher levels of GR in the chronic + acute condition with no change in GluA2/PKMζ. This pattern indicates that ST-housed animals are mobilizing GluA2 in a PKMζ-dependent manner as shown previously under learning conditions (Migues et al., 2010; Sebastian et al., 2013b; Braren et al., 2014). We propose that EE allows for enhanced mobilization of GR during chronic stress that reduces neuronal excitability and dampens the trafficking of both GluA2 and PKMζ in a calcium-dependent manner.

Glutamatergic transmission is critical for synaptic plasticity and maintaining membrane levels of GluA2 is important for memory (Migues et al., 2010; Sebastian et al., 2013b). Analysis of AMPA receptor trafficking shows that stabilizing the receptor blocks the CORT-induced trafficking which prevents LTP and restores synaptic plasticity (Zhang et al., 2013a). Functional imaging and histological studies of depressed patients show deficits in glutamatergic signaling (Yüksel and Öngür, 2010). The antidepressants fluoxetine and imipramine both increase phosphorylation of AMPA receptors, which presumably increase its stabilization on the membrane (Svenningsson et al., 2002; Du et al., 2007). These observations suggest that AMPA receptor stabilization is linked to the therapeutic impact of antidepressants involving monoaminergic and glutamatergic signaling (Manji et al., 2001; Berton and Nestler, 2006; Skolnick et al., 2009), a suggestion requiring further investigation.

## Author Contributions

PAS and VNL designed the experiments. RMZ fractionated brain samples and performed the western blots. BM, SHB and PAS conducted the statistical analyses. LMS conducted the CORT analysis. SBH and PAS wrote the manuscript. All authors approved the final manuscript for submission.

## Conflict of Interest Statement

The authors declare that the research was conducted in the absence of any commercial or financial relationships that could be construed as a potential conflict of interest.
